# Novel therapies in myeloid neoplasms show limited benefit and increased costs over 15 years of follow-up in Southern Finland

**DOI:** 10.1007/s00277-026-06863-y

**Published:** 2026-02-06

**Authors:** Theerin Lanamtieng, Anna Mervaala-Muroke, Laura Toivanen, Kimmo Porkka, Oscar Brück

**Affiliations:** 1 Hematoscope Lab, Comprehensive Cancer Center, Department of Oncology, Department of Clinical Chemistry, HUS Diagnostic Center, Helsinki University Hospital, University of Helsinki, Helsinki University Hospital, University of Helsinki, Tukholmankatu 8C, Helsinki, P.O. Box 705, FIN-00290 Finland; 2 Hematology Research Unit Helsinki, Department of Hematology, University of Helsinki, Helsinki University Hospital Comprehensive Cancer Center, Helsinki, Finland; 3 ICAN Digital Precision Cancer Medicine Flagship, University of Helsinki, Helsinki University Hospital Comprehensive Cancer Center, Helsinki, Finland

**Keywords:** Acute myeloid leukemia, Myelodysplastic syndromes, Myelofibrosis, Survivals, Cost analysis, Health economics

## Abstract

Over the past two decades, targeted therapies have been adopted in the treatment of myeloid neoplasms. However, their impact on overall survival (OS) and healthcare spending remains uncertain. We performed a comprehensive retrospective observational study on patients with acute myeloid leukemia (AML, *n* = 684), myelodysplastic syndrome (MDS, *n* = 899) and myelofibrosis (MF, *n* = 276) diagnosed between 2009 and 2023 in a large hospital district in Southern Finland. During this 15-year period, the median age increased across all cohorts. OS remained unchanged after adjusting for age, comorbidity index and risk score in AML (HR 0.77, 95% CI 0.59–1.02), MDS (HR 1.03, 95% CI 0.81–1.31), and MF (HR 0.70, 95% CI 0.45–1.08). However, progression-free survival (PFS) improved among AML patients. For subgroup analyses, OS and PFS improved in AML receiving high-dose chemotherapy, mostly due to better stratification to fit and unfit treatment regimens. In MDS and MF cohorts, no OS benefit was observed in any subgroup. Hospitalization and drug expenses were the primary components in all cohorts and increased notably in AML and MF patients due to novel treatments. Advances in clinical care and adoption of novel agents show limited benefit but have gradually increased their financial burden. Future research should prioritize less toxic, outpatient-based treatments to enhance outcome and cost-effectiveness.

**Supplementary Information:**

The online version contains supplementary material available at 10.1007/s00277-026-06863-y.

## Introduction

Over the last 20 years, therapeutic strategies of myeloid neoplasms have shifted from cytotoxic chemotherapy toward targeted approaches, guided by deeper understanding of molecular pathogenesis and risk stratification. This has led to improved survival, though challenges remain in high-risk and elderly populations [1–3]. These diseases are associated with substantial economic burdens due to their chronic nature, costly treatment requirements, and high rates of healthcare utilization [4–6].

In acute myeloid leukemia (AML), the standard of care for young patients without significant comorbidities (defined as fit) is intensive chemotherapy. However, the treatment landscape of AML has changed since 2017 with the incorporation of *FLT3* inhibitors, liposomal chemotherapy, and venetoclax-based regimens. Resistance and relapse remain major limitations, especially in high-risk patients such as those with *TP53* mutations or a complex karyotype [7], where durable remission is rarely achieved without allogeneic hematopoietic stem cell transplantation (alloHSCT). Several studies have demonstrated the survival improvement during the pre-targeted therapy era [8–13],likely driven by advances in supportive care. However, the evidence of survival improvement in recent years has been limited.

AML imposes a high upfront cost, particularly in patients who undergo intensive induction chemotherapy or alloHSCT. Hospitalization for induction and consolidation, management of complications and frequent transfusions contribute significantly to direct medical costs [4, 14]. The emergence of targeted agents, such as *FLT3* and *IDH* inhibitors, and venetoclax has added further costs [15].

In myelodysplastic syndromes (MDS), despite these advances, outcomes for patients with higher-risk MDS remain poor. AlloHSCT is the only curative treatment [16]. However, for transplant-ineligible patients, MDS becomes a chronic financial strain. Its chronic and transfusion-dependent forms accumulate long-term costs driven by blood transfusions, iron chelation therapy, and supportive care [17, 18]. Hypomethylating agents (HMA) typically administered for months to years, represents a major cost driver. The economic burden also increases after HMA failure [19].

In myelofibrosis (MF), the approval of ruxolitinib (RUX) and subsequent Janus kinase (JAK) inhibitors improved symptom, spleen burden management and survival [20–22]. Nevertheless, current therapies offer limited disease modification [23]. AlloHSCT remains a challenging due to timing and peri-transplantation management. MF patients face a long-term economic burden due to the chronic use of JAK inhibitors [6].

Across all three diseases, targeted therapies may have partly improved outcomes but also escalated treatment-related costs. Hospitalizations, transfusions, long-term drug administration, remain major contributors to direct healthcare expenditure. Evaluating the cost-effectiveness of novel therapies is critical to ensure equal access. However, this type of analysis requires comprehensive, high-quality real-world data and transparent information on healthcare unit costs, which are rarely available even in most developed countries due to complex, proprietary and siloed information technology systems.

## Methods

### Patients

We conducted a retrospective cohort study to evaluate trends in overall survival (OS) and healthcare costs among patients diagnosed with myeloid neoplasms, with a particular focus on the impact of the diagnostic period. The study included adult patients (≥ 16 years old) diagnosed at the Helsinki and Uusimaa Hospital District (HUS), Finland’s largest hospital district covering 1.7 million inhabitants with a network of almost 30 hospitals. We included all AML, MDS and MF patients diagnosed between January 2009 and December 2023 in the hospital district. Patients with acute promyelocytic leukemia were excluded. Participants were grouped into three 3-year diagnostic cohorts based on their year of diagnosis: 2009–2013, 2014–2018, and 2019–2023.

### Clinical data

All clinical care records, including demographic characteristics, diagnostic information, risk stratifications, treatment regimens, and survival outcomes, were stored at the HUS Datalake – a secure, cloud-based environment designed for secondary health data analysis.

All other information were retrieved (e.g. clinical visits, laboratory tests) and encoded (e.g. risk scores, survival times) with an in-house data extraction pipeline. We used the European LeukemiaNet (ELN) 2022 risk stratification system for AML, the IPSS-R (Revised International Prognostic Scoring System) for MDS, and the DIPSS (Dynamic International Prognostic Scoring System) for MF. The Charlson Comorbidity Index (CCI) [24] was used to assess baseline comorbidity burden.

Healthcare resource utilization and costs were analyzed across six categories: imaging, outpatient visits, procedures, drug costs, laboratory testing, and inpatient days. These cover all healthcare resource use at the HUS hospital district for the treatment of myeloid neoplasms and other severe medical conditions. Procedural costs covered major interventions, including alloHSCT, transfusions, and surgeries. Drug costs were estimated for disease-directed therapies. Costs for unrelated medications were excluded. Drug-level cost data were estimated based on median dose and treatment duration per regimen. We retrieved individual drugs at various times from the HUS pharmacy database and the national Pharmaceuticals Pricing Board.

### Statistical analyses

Kaplan-Meier survival curves were generated to illustrate trends in OS and progression-free survival (PFS) across diagnostic periods. OS was defined as the time from diagnosis to death or last follow-up, while PFS was defined as the time from diagnosis to disease progression, relapse, or death, whichever occurred first. Due to limitations in data availability, PFS was assessed only in the AML cohort. To evaluate the impact of diagnostic period on OS and PFS while adjusting for CCI and risk stratification, multivariable Cox proportional hazards models were employed. Subgroup analyses were performed using Kaplan-Meier curves and the log-rank test.

For economic analysis, mean costs were calculated. Multivariable linear regression models were used to compare costs across diagnostic periods, adjusting for follow-up time and CCI. We used Pearson’s Chi-squared test and Fisher’s exact test to compare categorical variables. We used Kruskal-Wallis rank sum test to compare continuous variables. To assess whether continuous variables exhibited a monotonic trend across ordered groups, we visualized linear trend line from linear regression model and used Jonckheere-Terpstra test. To assess whether there was a statistically significant linear trend in proportions across the diagnostic periods, we applied the Cochran-Armitage Trend Test. We conducted all analyses using R (version 4.4.3).

### Subgroup analyses

Subgroup analyses were performed separately within the AML, MDS, and MF cohorts to examine potential heterogeneity in survival outcomes. In the AML cohort, OS and PFS subgroups were defined by gender, age (< 70 vs. ≥70), and receipt of high-dose chemotherapy (HDC). In the MDS cohort, OS subgroups were defined by gender, age (< 70 vs. ≥70), and treatment with hypomethylating agents. For the MF cohort, OS subgroups were based on gender, age (< 70 vs. ≥70), and treatment with JAK inhibitors.

For cost outcomes, subgroup analyses were guided by disease-specific risk classifications reflecting differences in treatment approaches and associated healthcare utilization. In the AML cohort, risk categories were based on the ELN classification. In the MDS cohort, risk categories were derived from IPSS-R and dichotomized into higher-risk (IPSS-*R* > 3.5) and lower-risk (IPSS-*R* ≤ 3.5) groups, reflecting clinically distinct treatment strategies that impact costs. In the MF cohort, risk categories were defined using DIPSS and grouped into higher-risk (high and intermediate-2) and lower-risk (intermediate-1 and low) categories, which correspond to differences in disease burden and treatment modalities influencing healthcare costs.

### Ethical consideration

The study adhered to the Declaration of Helsinki and to European and national data privacy regulations. The use of clinical data was approved by the institutional research board (study permit HUS/179/2024). Personal data in this registry is processed in accordance with Article 9(2) of the General Data Protection Regulation and Act on the Secondary Use of Health and Social Data, which permit the use of personal health data for scientific research purposes subject to appropriate safeguards without notifying each individual separately. However, individuals have the right to prohibit the use of their data.

## Results

### Age and comorbidity in patients with myeloid neoplasms

In this study, we evaluated advances in treatment patterns of myeloid neoplasms and their translation to outcomes and healthcare expenditure. The analyses were conducted using the HUS Datalake, a secure, cloud-based repository comprising comprehensive medical data from over 3.5 million patients.

As any improvements in OS at the population level or supportive care could represent a confounding factor, we examined AML (*n* = 684), MDS (*n* = 899) and MF (*n* = 276) in parallel. The median follow-up duration was 15.4 months for AML, 31.8 months for MDS, and 44.8 months for MF. Baseline characteristics for each group are detailed in Table 1 (AML), Table 2 (MDS), and Table 3 (MF). Median age increased over time across all cohorts. The CCI decreased over time in patients with AML, remained stable in those with MDS, and increased in MF (Supplementary Fig. 1). However, among AML patients treated with HDC, age and CCI had a significantly decreasing trend (Supplementary Fig. 2).

**Table 1:** Baseline characteristics of the acute myeloid leukemia cohort

Characteristic	2009–2013	2014–2018	2019–2023	*P*-value^1^
*N* = 684	*N* = 183	*N* = 223	*N* = 278	
Female (%)	93/183 (51)	120/223 (54)	148/278 (53)	0.8
Male (%)	90/183 (49)	103/223 (46)	130/278 (47)	
Median age [25–75%]^2^	65 [52–75]	68 [54–76]	70 [55–79]	0.028
Median follow up time (year) [25–75%]^2^	1.1 [0.2–9.8]	1.5 [0.4–7.0.4.0]	1.3 [0.3–3.0.3.0]	0.017
ELN classification (%)				< 0.001
- Favorable	34/130 (26)	49/188 (26)	70/246 (28)	
- Intermediate	48/130 (37)	51/188 (27)	38/246 (15)	
- Adverse	48/130 (37)	88/188 (47)	138/246 (56)	
- Unknown	53	35	32	
Median CCI [25–75%]^2^	3 [2–5]	3 [2–5]	3 [1–5]	0.029
AlloHSCT (%)	48/183 (26)	54/223 (24)	71/278 (26)	0.9
Treatment (%)				
- HDC	126/183 (69)	113/223 (51)	120/278 (43)	< 0.001
Total cycle of HDC				0.5
▪ 1–2	50/126 (40)	44/113 (39)	40/120 (33)	
▪ 3–5	75/126 (60)	69/113 (61)	80/120 (67)	
▪ Unknown	1/126 (1)	0/113 (0)	0/120 (0)	
Cycle of induction				0.3
▪ 1	99/126 (79)	85/113 (75)	84/120 (71)	
▪ 2–3	26/126 (21)	28/113 (25)	35/120 (29)	
▪ Unknown	1/126 (1)	0/113 (0)	1/120 (1)	
Cycle consolidation				0.5
▪ 0–1	58/126(46)	57/113 (50)	51/120 (43)	
▪ 2–3	67/126 (21)	56/113 (50)	69/120 (58)	
▪ Unknown	1/126 (1)	0/113 (0)	0/120 (0)	
- HMA monotherapy	5/183 (3)	51/223 (23)	42/278 (15)	< 0.001
- HMA + VEN	< 3/183 (0)	5/223 (2)	48/278 (17)	< 0.001
- FLT3 inhibitor	< 3/183 (1)	6/223 (3)	23/278 (8)	< 0.001
- GO	3/183 (2)	6/223 (3)	16/278 (6)	0.045
- Liposomal daunorubicin and cytarabine	< 3/183 (0)	< 3/223 (1)	11/278 (4)	0.001
Etiology (%)				
- De-novo	124/183 (68)	148/223 (66)	206/278 (74)	0.011
- Myelodysplastic-related	44/183 (24)	51/223 (23)	36/278 (13)	
- Therapy-related	15/183 (8)	24/22 (11)	36/278 (13)	

^1^Pearson’s Chi-squared test, Kruskal-Wallis rank sum test, Jonckheere-Terpstra test, ^2^Interquartile range

Abbreviation: *ELN* European LeukemiaNet, *CCI* Charlson Comorbidity Index, *AlloHSCT* Allogeneic hematopoietic stem cell transplantation, *HDC* High-dose chemotherapy, *HMA* Hypomethylating agents, *VEN* Venetoclax, *GO* Gemtuzumab Ozogamicin

**Table 2:** Baseline characteristics of the myelodysplastic syndromes cohort

Characteristic	2009–2013	2014–2018	2019–2023	*P*-value^1^
*N* = 899	*N* = 286	*N* = 272	*N* = 341	
Female (%)	122/286 (43)	134/272 (49)	119/341 (35)	0.002
Male (%)	164/286 (57)	138/272 (51)	222/341 (65)	
Median age [25–75%]^2^	73 [65–81]	75 [66–82]	75 [68–82]	0.002
Median follow up time (year) [25–75%]^2^	3.1 [1.0–8.8.0.8]	3.5 [1.3–6.9]	2.2 [1.2–3.4]	< 0.001
IPSSR (%)				0.3
- Very low	30/212 (14)	40/193 (21)	37/262 (14)	
- Low	109/212 (51)	84/192 (44)	134/262 (51)	
- Intermediate	37/212 (17)	40/193 (21)	44/262 (17)	
- High	27/212 (14)	26/193 (13)	34/262 (13)	
- Very high	9/212 (4)	3/193 (2)	13/163 (5)	
- Unknown	74	79	79	
Median CCI [25–75%]^2^	4 [2–5]	4 [3–5]	4 [3–6]	0.12
AlloHSCT (%)	28/286 (10)	33/272 (12)	27/341 (8)	0.2
Treatment (%)				
- HMA	81/286 (28)	79/272 (38)	125/341 (37)	0.032
- Erythropoietin	130/286 (45)	137/272 (50)	160/341 (47)	0.5
Therapy-related MDS (%)	43/286 (15)	33/272 (12)	68/341 (20)	0.028

^1^Pearson’s Chi-squared test, Kruskal-Wallis rank sum test, Jonckheere-Terpstra test, ^2^Interquartile range

Abbreviation: *IPSSR* Revised International Prognostic Scoring System, *CCI* Charlson Comorbidity Index, *alloHSCT* Allogeneic hematopoietic stem cell transplantation, *HDC* High-dose chemotherapy, *HMA* Hypomethylating Agents

**Table 3:** Baseline characteristics of the myelofibrosis cohort

Characteristic	2009–2013	2014–2018	2019–2023	*P*-value^1^
*N* = 276	*N* = 82	*N* = 83	*N* = 111	
Female (%)	32/82 (39)	46/83 (55)	45/111 (41)	0.058
Male (%)	50/82 (61)	37/83 (45)	66/111 (59)	
Median age [25–75%]^2^	67 [60–75]	70 [64–76]	74 [63–79]	0.009
Median follow up time (year) [25–75%]^2^	5.5 [2–11.6.6]	5.4 [2.0–8.1.0.1]	2.4 [1.4–4.1]	< 0.001
DIPSS (%)				0.4
- Low	13/78 (17)	7/81 (9)	13/109 (12)	
- Intermediate-1	40/78 (51)	39/81 (48)	54/109 (50)	
- Intermediate-2	20/78 (26)	30/81 (37)	30/109 (28)	
- High	5/78 (6)	5/81 (6)	12/109 (11)	
- Unknown	4	2	3	
Median CCI [25–75%]^2^	3 [1–4]	3 [2–4]	4 [2–5]	0.014
AlloHSCT (%)	10/82 (12)	6/83 (7)	8/111 (7)	0.4
JAK inhibitors (%)	12/82 (15)	27/83 (33)	33/111 (30)	0.017
Etiology (%)				0.006
- Primary	51/82 (62)	35/83 (42)	55/111 (50)	
- Post-ET	13/82 (16)	16/83 (19)	33/111 (30)	
- Post-PV	18/82 (22)	32/83 (39)	23/111 (21)	

^1^Pearson’s Chi-squared test, Kruskal-Wallis rank sum test, Jonckheere-Terpstra test, ^2^Interquartile range

Abbreviation: *DIPSS* Dynamic International Prognostic Scoring System, *CCI* Charlson Comorbidity Index, *AlloHSCT* Allogeneic hematopoietic stem cell transplantation, *HDC* High-dose chemotherapy, *JAK* Janus kinase, *ET* essential thrombocythemia, *PV* polycythemia vera

### Proportion of risk classes and treatments in myeloid neoplasms overtime

In AML, the proportion of patients with adverse-risk ELN classification increased in recent years possibly due to an aging population and higher incidence of t-AML following improved treatment of other hematological and solid tumors (Table 1 and Supplementary Fig. 3A). Similarly, the proportion of patients with t-MDS has risen over time (Table 2). In MF, cases of secondary MF evolving from essential thrombocythemia have also become more frequent in recent years (Table 3).

Next, we examined therapy at different times. The use of HDC in AML has decreased (Table 1 and Supplementary 3B). Despite a decreasing trend, there were no differences in the distribution of total cycle of HDC, induction cycles and consolidation cycles by diagnostic periods (Table 1). The proportion of patients undergoing alloHSCT remained stable across diagnostic periods. When analyzed by ELN risk classification, alloHSCT utilization was highest in the intermediate-risk group (40.2%), followed by the favorable-risk group (32.0%) and the adverse-risk group (23.4%).

for less intensive treatment, we pooled azacitidine/decitabine monotherapy and their combination with venetoclax into HMA-based regimens. From 2009 to 2023, there has been an increase in HMA-based regimens usage (Table 1 and Supplementary 3B). While HMA monotherapy became common in patients diagnosed 2014–2018, its use was partly replaced by the combination of HMA and venetoclax in 2019–2023 (Table 1).

In MDS, the distribution of IPSS-R scores has remained stable (Table 2 and Supplementary Fig. 3C). Consistently, the use of erythropoiesis-stimulating agents has not varied (Supplementary Fig. 3D). In addition, the use of HMA and alloHSCT peaked in the 2014–2018 cohort (Supplementary Fig. 2D).

In MF, the distribution of DIPSS risk classes has remained stable. (Table 3 and Supplementary Fig. 3E). JAK inhibitor use has increased, corresponding to the proportion of patients with an intermediated-II or high-risk class at diagnoses (Table 3 and Supplementary Fig. 2F). Instead, the proportion of alloHSCT has remained at a rate of 7–12% (Table 3 and Supplementary Fig. 3F).

### OS and PFS improved in AML with HDC

**Fig. 1:**
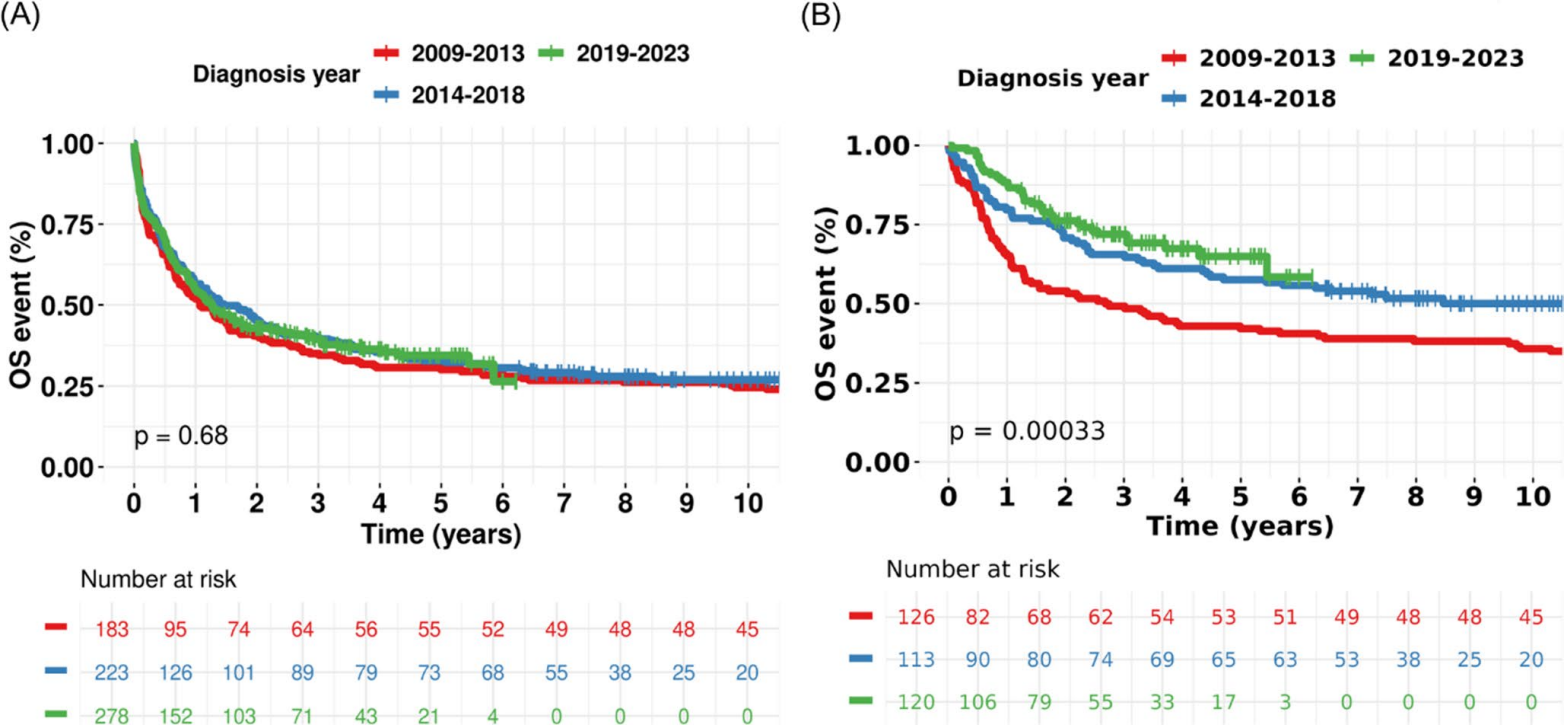
Kaplan–Meier curve with log-rank *p*-value for overall survival across diagnosis periods in acute myeloid leukemia (AML). (**A**) Whole AML cohort. (**B**) Treated with high-dose chemotherapy (HDC)

Given the adoption of multiple novel drugs, we interrogated next their impact on long-term outcomes, adjusting for CCI and risk stratification. We did not observe changes in OS across diagnostic periods for AML (Figs. 1A; adjusted HR 0.77, 95% CI 0.59–1.02), MDS (adjusted HR 1.03, 95% CI 0.81–1.31), or MF (adjusted HR 0.70, 95% CI 0.45–1.08). PFS was available in the AML cohort (Supplementary Fig. 4). In unadjusted analysis, the difference in PFS across diagnostic periods was not statistically significant (log-rank *p* = 0.06). However, after adjusting for CCI and ELN risk stratification, the diagnostic period was associated with improved PFS (adjusted HR 0.74, 95% CI 0.57–0.93).

In subgroup analysis of AML, Kaplan-Meier analysis showed statistically significant improvement of OS and PFS in AML patients receiving HDC (log-rank *p* < 0.001, Fig. 1B and Supplementary Fig. 4B). In patients ≥ 70 years old, the improvement in PFS was observed (log-rank *p* = 0.002, Supplementary Fig. 4E). The improvements were not observed among gender or other age groups (Supplementary Fig. 5). Early mortality rate of patients with HDC within first 6 weeks decreased over the three diagnostic periods: 7.1% (9/126) in 2009–2013, 3.5% (4/113) in 2014–2018, and 0.8% (1/120) in 2019–2023 (*p* = 0.01, Cochran-Armitage trend test).

**Fig. 2:**
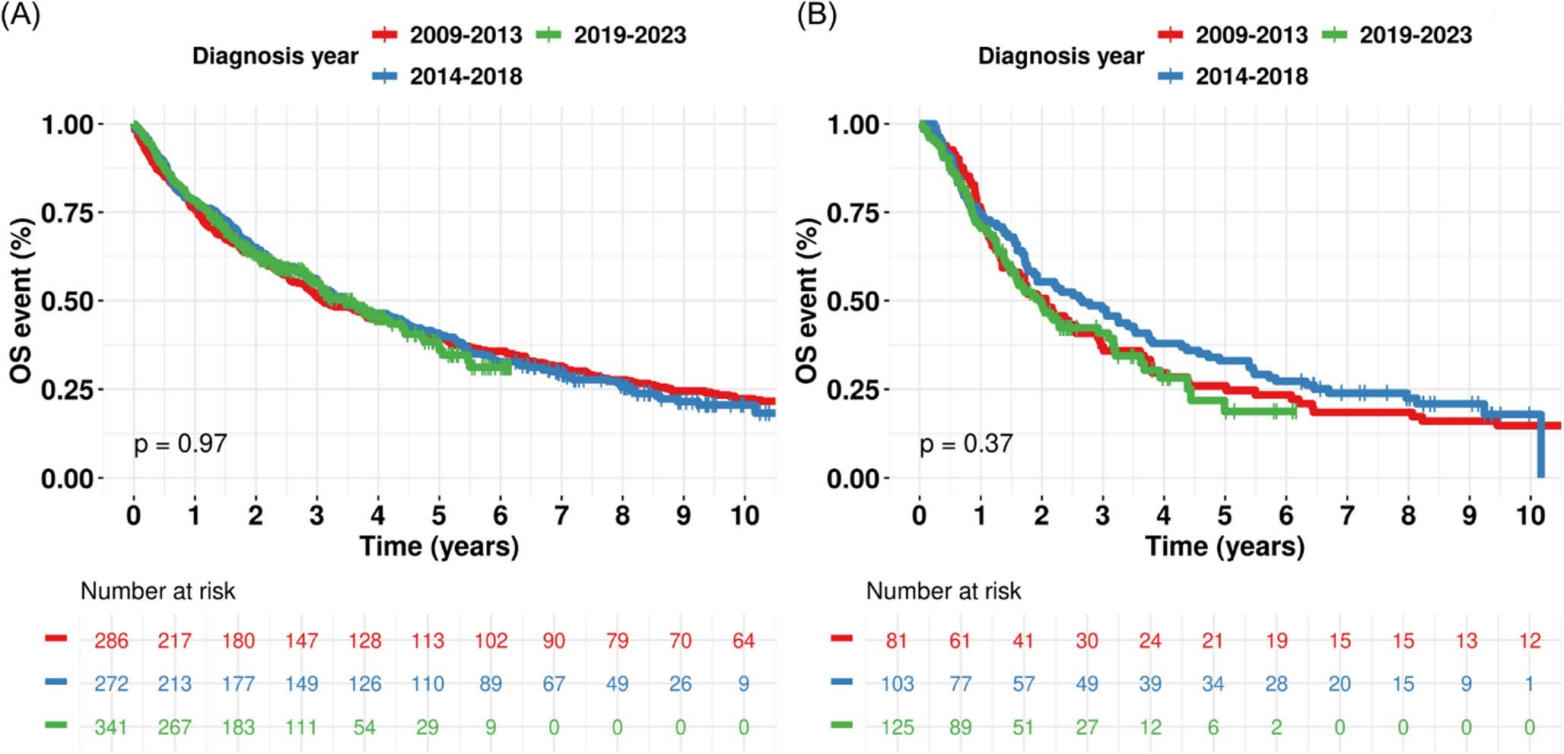
Kaplan–Meier curve with log-rank *p*-value for overall survival across diagnosis periods in myelodysplastic syndrome (MDS). (**A**) Whole MDS cohort. (**B**) Treated with hypomethylating agents

**Fig. 3:**
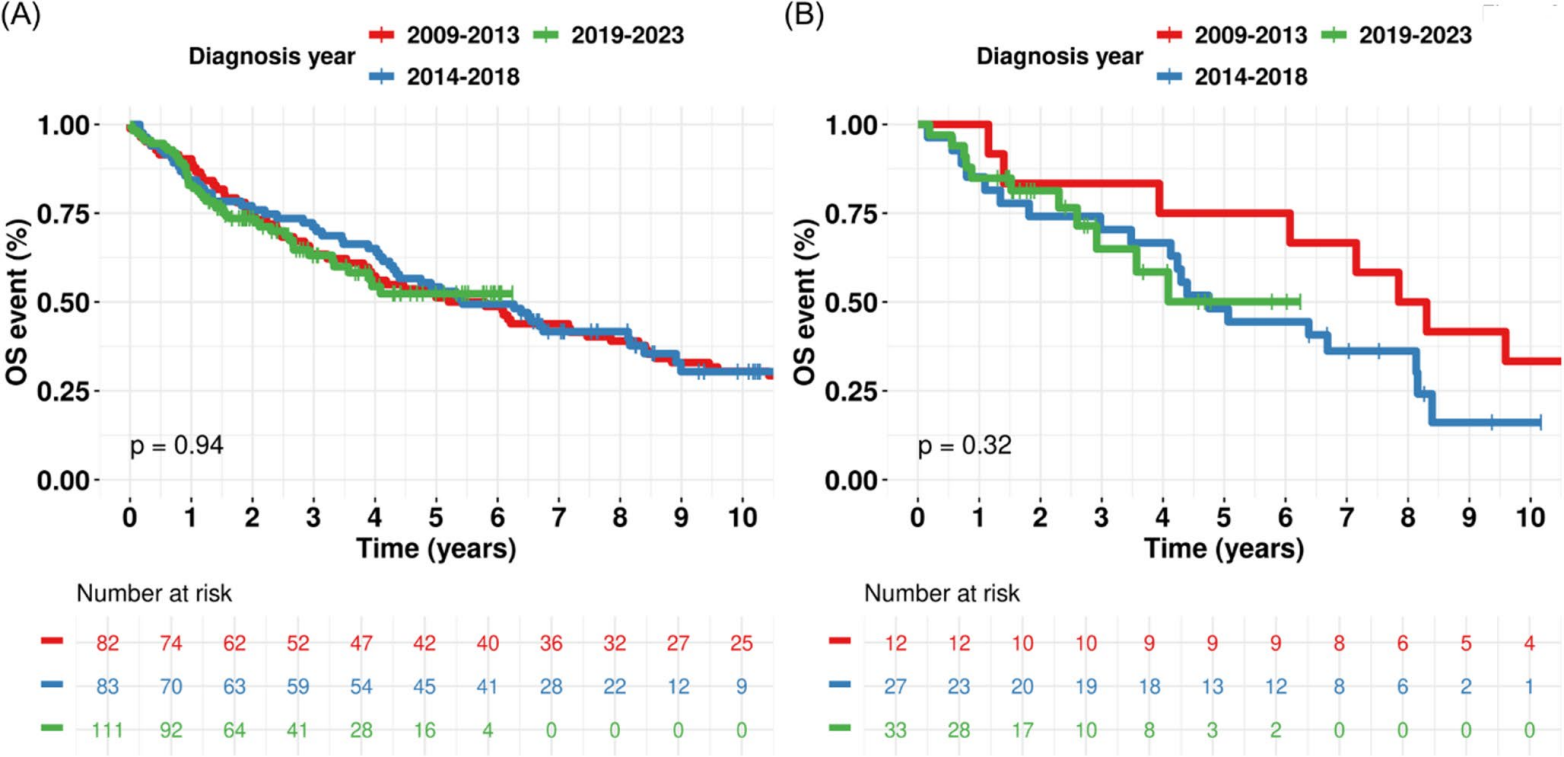
Kaplan–Meier curve with log-rank *p*-value for overall survival across diagnosis periods in myelofibrosis (MF). (**A**) Whole MF cohort. (**B**) Treated with JAK inhibitors

In the MDS, the whole cohort (Fig. 2A), subgroup analyses by sex, age group (Supplementary Fig. 6), and treatment with HMA (Fig. 2B) did not demonstrate significant improvements in OS. Similarly, in the MF, the whole cohort (Fig. 3A), subgroup analyses by sex, age group (Supplementary Fig. 7), and treatment with JAK inhibitors (Fig. 3B) did not show significant survival benefits.

### The cost of treating myeloid neoplasms

Because patients diagnosed between 2009 and 2013 had a longer follow-up period than those in other groups, we minimized the risk of follow-up bias by focusing our analysis on the first year after diagnosis. Importantly, this period typically captures the majority of costs associated with diagnostic workup, hospitalization, and initiation of intensive treatment.

**Fig. 4:**
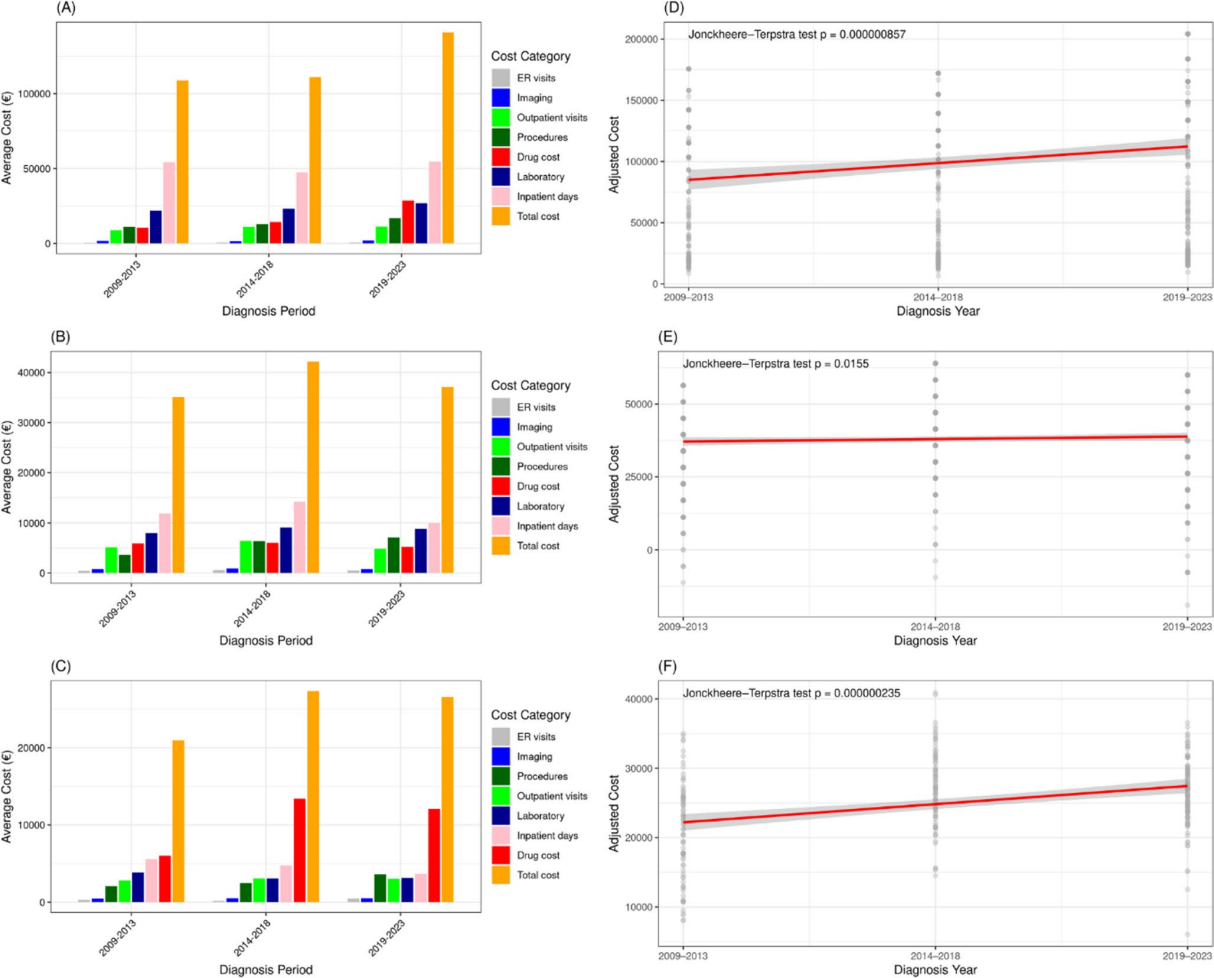
Healthcare resource use by disease groups and diagnosis periods One-year mean costs by healthcare resource category (left panel) and scatter plot with linear regression line of adjusted total costs (right panel) by diagnostic periods in patients with (**A**, **B**) acute myeloid leukemia, (**C**, **D**) myelodysplastic syndrome, (**E**, **F**) myelofibrosis

The mean one-year cost per patient across all categories for AML MDS and MF are shown in Supplementary Table 1. For the AML cohort, inpatient care was the largest cost driver (Fig. 4A). Inpatient care costs declined in 2014–2018 coinciding with the drop in HDC. We observed that the duration of hospitalization in the AML cohort remained stable over the years, but there was a significant increase in the frequency of inpatient admissions. (Supplementary Fig. 8). Total medical costs were highest in the intermediate-risk group, followed by the favorable-risk group, and lowest in the adverse-risk group (Supplementary Fig. 9A). Costs increased in all risk groups from 2009 to 2013 to 2019–2023.

For the MDS cohort, inpatient care increased up to 2014–2018 but decreased followingly (Supplementary Table 1). While these continued to dominate expenses, we could not discern similar increase due to outpatient or emergency costs (Fig. 4B). Higher-risk MDS patients incurred greater total medical costs than lower-risk patients (Supplementary Fig. 9B).

The cost profile of MF patients resembled that of MDS patients except for higher outpatient and emergency care costs (Supplementary Table 1). In the MF cohort, drug expenses represented the major cost component in recent years and their costs have continued to increase due to higher use of JAK inhibitors (Fig. 4C). Patients classified as higher-risk according to DIPSS had higher total medical costs than those in lower-risk categories (Supplementary Fig.  9C).

Next, we interrogated how the cost profile of most intensively treated patients of each disease group has evolved. The expenses of HDC-treated AML patients have increased (Supplementary Table 2). For comparison, changes in HMA-treated MDS patients and JAK inhibitor-treated MF patients have remained modest (Supplementary Table 2).

Given the changes in patient age and comorbidities, and therefore treatment strategies, we adjusted mean one-year total costs for patient age and CCI. We observed that AML-related costs showed an uptick in recent years, likely due to the introduction of novel therapies. In MDS, mean costs peaked during 2014–2018, coinciding with increased HMA use and a higher frequency of HSCT, which contributed to increased inpatient admissions. For MF, the highest total costs were also observed during 2014–2018, aligning with higher proportion of JAK inhibitors usage. When analyzed with a linear trend line, all three disease cohorts showed a significant increasing trend Figure (4D-6 F).

## Discussion

By spanning a 15-year period at a large public hospital district, this study presents one of the most comprehensive real-world analyses combining outcomes and healthcare costs in patients across myeloid neoplasms. Drawing from an extensive cohort of almost 1,900 patients with AML, MDS, and MF, we analyzed records for every hospital visit, medical procedures, diagnostic tests and treatments.

A striking observation is the progressive increase in patient age over time. This aligns with national demographic shifts in Finland, which has one of the most rapidly aging populations in Europe. According to Statistics Finland, individuals aged 65 years and older now constitute nearly 24% of the total population in 2024 [25]. The proportion of patients receiving HDC decreased over time due to the increase in elderly patients shifting treatment strategies toward lower-intensity regimens such as HMA or venetoclax-based combinations in recent years [26–29].

Despite the rising burden of age, known to negatively impact survival outcomes, OS has remained stable in AML across diagnostic periods with improvement of PFS. Among AML patients who received HDC, we observed a significant OS and PFS benefit in recent years. The improvement of fit patients mirrored findings from a large Swedish population-based cohort [30] and a single-center study from France [13]. However, our data suggest that the apparent improvement in this subgroup reflects simply a more accurate distinction of patients by fitness, as indicated by lower age and comorbidities.

In patients older than 70, the improvement in PFS without a corresponding OS benefit suggests that the therapy provides better immediate disease control. Interestingly, a recent study from a nationwide U.S. cohort showed improved survival outcomes among AML patients aged > 70 years in the post-novel therapy era (2019–2022), with approximately 40% of patients receiving venetoclax-based therapies [26]. In contrast, among patients in the same age group in our cohort, OS did not improve significantly. This discrepancy could be explained as only 20% of our corresponding study population received venetoclax-based treatment. As the survival benefit of HMA+venetoclax over HMA monotherapy at 24 months is ~ 20% higher suggests our study is underpowered to detect such as modest benefit [31].

Survival outcomes in MDS did not improve over time, which is not surprising given the lack of new disease-modifying therapies in the past two decades [32]. We reasoned that advances in supportive care may have stabilized OS despite increasing age [33]. When examining large population-based studies comparing survival outcomes from the 2000 s to the 2010 s, the OS of MDS has not been reported to improve [34, 35].

For MF, although some studies have reported improved outcomes in recent years, our study did not demonstrate any OS improvement. Masarova et al. [36] reported a significant improvement in OS in 2011–2020 compared to 2000–2010. However, both patient age and splenic burden were higher in the later period. Notably, the proportion of patients receiving RUX was substantially higher in their study (63% during 2011–2020) compared to our cohort (33% during 2014–2018). These discrepancies highlight the importance of assessing advances in clinical management in public healthcare to exclude bias caused by treatment availability and patient selection.

In our study, inpatient days remained the largest contributor for AML, aligning with previous research conducted in both European and North American cohorts [37–39]. This reflects the intensive nature of AML treatment. When comparing the total mean 1-year cost for AML patients to other European countries, our findings aligned with a report from the Netherlands [40, 41].

The trend in total costs of AML cohort increased in the most recent period (2019–2023) driven by the introduction and broader use of novel targeted therapies, such as venetoclax-based regimens. These agents, while effective, come with high drug acquisition costs. Furthermore, laboratory testing costs peaked during this period reflecting increased adoption of advanced diagnostic technologies such as molecular profiling to guide personalized treatment.

In the earlier diagnostic periods (2009–2018), the inpatient cost declined. As a result, low-intensity regimens became more common and even home-based consolidation treatments were initiated. However, inpatient costs increased again during 2019–2023. This period included a higher proportion of older patients, as well as increased use of HMA + venetoclax and a greater number of healthcare encounters, which may partially explain the rise in inpatient expenditures.

In contrast to AML and MDS, drug costs were the predominant component of total medical expenses in MF. The total cost was highest in the 2014–2018 cohort, corresponding to increased adoption of JAK inhibitors. This pattern is consistent with previous studies reporting that JAK inhibitors are the principal cost driver in MF management [6, 42, 43].

When examining total medical costs in relation to risk classification, AML patients in the intermediate-risk group incurred the highest expenditures, likely due to more intensive treatment strategies and a greater proportion of undergoing alloHSCT. The adverse-risk group experienced likely higher proportion of refractory disease and early mortality, which could limit costs. In MDS and MF, higher-risk categories were similarly associated with increased costs, reflecting the greater need for aggressive therapies and supportive care as disease severity advances.

We acknowledge that this study has certain limitations. As a single-center retrospective analysis in a country with public healthcare, our findings may not generalize to countries with different reimbursement models, drug accessibility, or clinical practices. We did not include costs of medications related to comorbidities as we expected these to accumulate significantly lower expenses to costs of treating myeloid neoplasms. Due to the lack of exact drug-level cost data, medication expenses were estimated based on standard median doses and treatment durations. However, all other cost categories were derived from actual charges recorded in the hospital’s electronic medical records, enhancing the reliability of those components. Indirect costs, such as lost productivity and caregiver burden, were not captured in this analysis.

Another limitation of the cost analysis is that we analyzed costs only during the first year after diagnosis. Thus, we did not capture long-term healthcare use, which may vary by risk group, treatment decisions, comorbidities, or other health events that are not related to the myeloid neoplasms. However, focusing on the first year also has an important benefit. This period includes the diagnostic workup, initial hospitalization, and the start of treatment, which constitute a critical and comparable section of the cost analysis, and these are most directly related to the disease. As a result, the total economic burden over the full disease course is likely underestimated.

Finally, although we adjusted for major confounding factors such as comorbidity burden, risk stratification and follow-up duration, unmeasured variables could still influence survival and cost outcomes, and causality cannot be established in this observational design.

This study provides a comprehensive real-world evaluation of survival outcomes and healthcare costs for myeloid neoplasms in almost two decades. Despite increasing age, survival outcomes in AML, MDS and MF remained stable, whereas the economic burden increased particularly in AML patients due to escalating drug costs. These findings highlight the importance of balancing clinical efficacy with cost-effectiveness, especially in aging populations.

## Supplementary Information

Below is the link to the electronic supplementary material.

Supplementary Material 1

## Data Availability

The original data can be accessed with an appropriate study and data permit from the Helsinki University Hospital or the Finnish Social and Health Data Permit Authority Findata. The curated datasets used in this study can be accessed upon reasonable request from the corresponding authors.
