# Supplementary material for: Novel therapies in myeloid neoplasms show limited benefit and increased costs over 15 years of follow-up in Southern Finland
Source: Ann Hematol. 2026 Feb 6;105(3):94. doi: 10.1007/s00277-026-06863-y (PMC12881082; doi:10.1007/s00277-026-06863-y)

Supplementary table 1. One-Year Mean Costs by Category and Patient (€)

	2009-2013	2014-2018	2019-2023
**Acute Myeloid Leukemia**			
Emergency visits	400	600	600
Imaging	1600	1500	1900
Outpatient visits	8800	11000	11290
Procedures	11100	12900	17000
Drug cost	10600	13400	21200
Laboratory	21900	23100	26900
Inpatient days	54400	47500	54600
Total cost	108900	110000	133500
**Myelodysplastic Syndromes**			
Emergency visits	500	600	500
Imaging	800	900	800
Outpatient visits	5100	6400	4800
Procedures	3600	6400	7100
Drug cost	5900	6000	5200
Laboratory	8000	9100	8900
Inpatient days	11900	14200	10000
Total cost	35100	42200	37100
**Myelofibrosis**			
Emergency visits	300	200	500
Imaging	500	500	500
Outpatient visits	2800	3100	3000
Procedures	2100	2500	3600
Drug cost	6000	13400	12100
Laboratory	3800	3100	3100
Inpatient days	5600	4800	3700
Total cost	21000	27300	26500

Supplementary table 2. One-Year Mean Costs by Category by treatment subgroup within each disease cohort (€)

	2009-2013	2014-2018	2019-2023
**Acute Myeloid Leukemia with High Dose Chemotherapy**			
Emergency visits	400	400	500
Imaging	2200	2100	3100
Outpatient visits	11200	14100	17000
Procedures	15300	21400	31800
Drug cost	14200	22800	44900
Laboratory	29200	36200	47200
Inpatient days	74500	76200	95900
Total cost	147100	173200	240500
**Myelodysplastic Syndromes with Hypomethylating agents**			
Emergency visits	700	900	900
Imaging	1300	1300	1300
Outpatient visits	11200	12700	9700
Procedures	6500	12100	14100
Drug cost	10800	8500	7500
Laboratory	14700	14700	17000
Inpatient days	21700	24100	21600
Total cost	66900	72600	72100
**Myelofibrosis with JAK inhibitors**			
Emergency visits	300	300	400
Imaging	200	700	700
Outpatient visits	1300	1300	1300
Procedures	4100	4000	3800
Drug cost	40700	40700	40700
Laboratory	4800	3800	4200
Inpatient days	7300	4100	4400
Total cost	59900	55000	58500

Supplementary Figure 1. Patient age and comorbidity profiles by disease. Median of age at diagnosis (left panel) and linear regression line of Charlson Comorbidity Index (CCI) (right panel) for patients with (A-B) acute myeloid leukemia, (C-D) myelodysplastic syndrome, and (E-F) myelofibrosis patients.

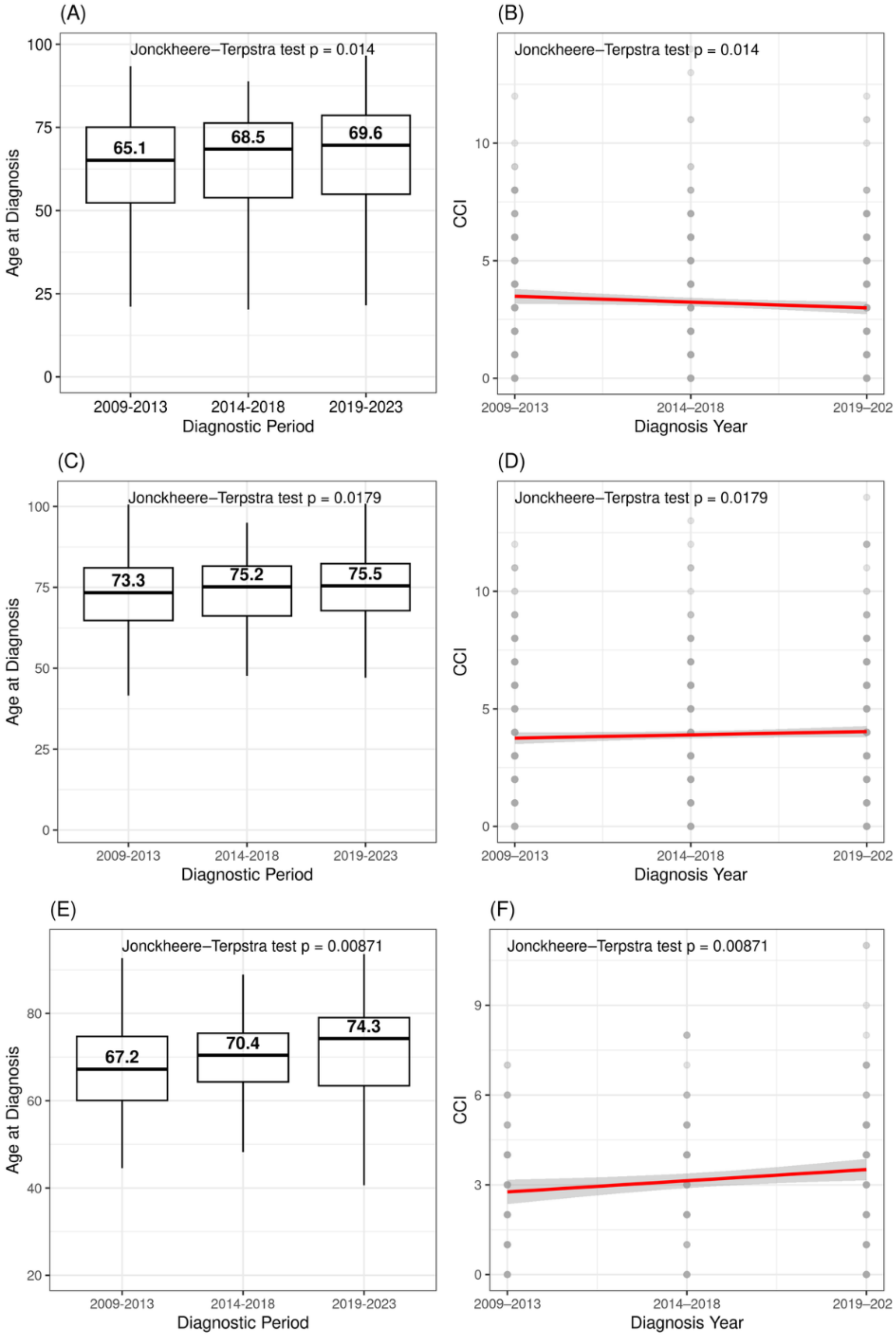

Supplementary Figure 2. Patient demographics in acute myeloid leukemia patients treated with high dose chemotherapy.

(A) Median of age at diagnosis
(B) linear regression line of Charlson Comorbidity Index (CCI) among acute myeloid leukemia patients with high dose chemotherapy.

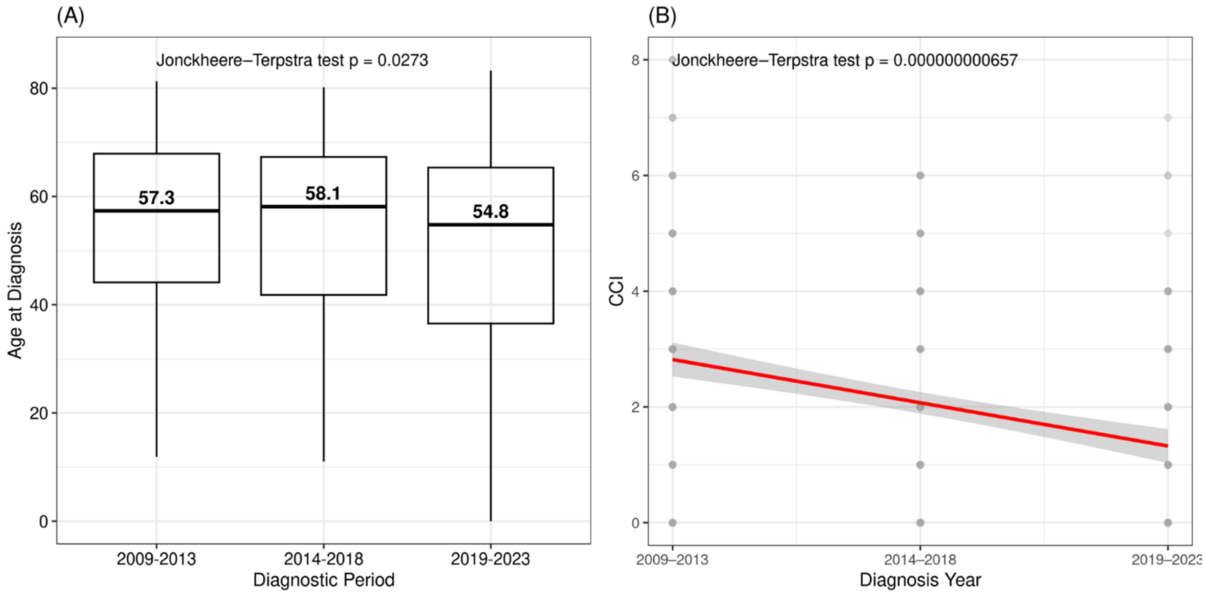

Supplementary Figure 3. Risk scores and treatment trends.

Proportions of (A-B) acute myeloid leukemia, (C-D) myelodysplastic syndrome and (E-F) myelofibrosis patients by risk classes and common treatment regimens by diagnostic periods.

Abbreviations: European LeukemiaNet (ELN), Revised International Prognostic Scoring System (IPSSR) and Dynamic International Prognostic Scoring System (DIPSS).

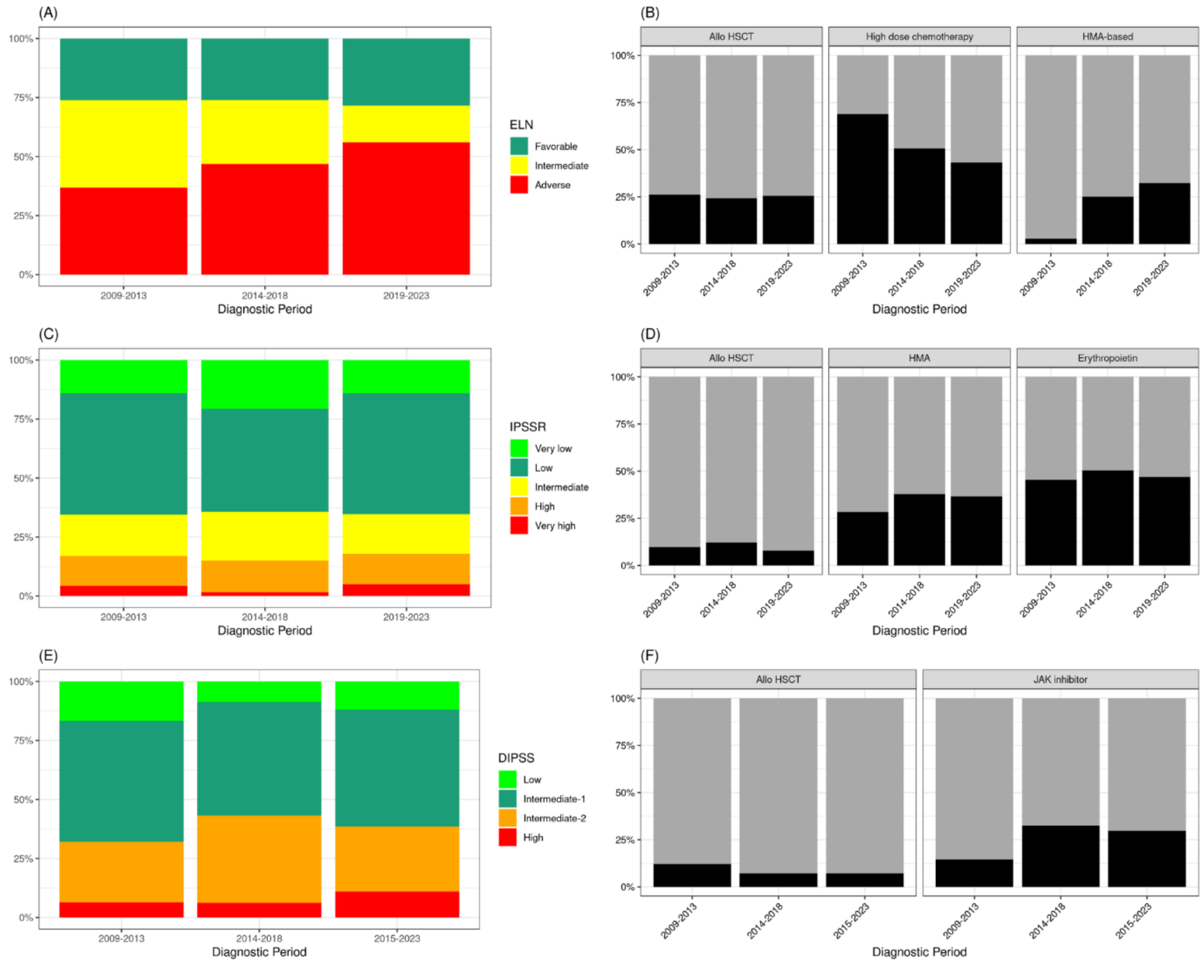

Supplementary Figure 4. Kaplan–Meier curve with log-rank p-value for progression-free survival across diagnosis in acute myeloid leukemia (AML)

(A) Whole AML cohort
(B) Treated with high-dose chemotherapy
(C) Female
(D) Male
(E) Age 16–69 years
(F) Age ≥70 years

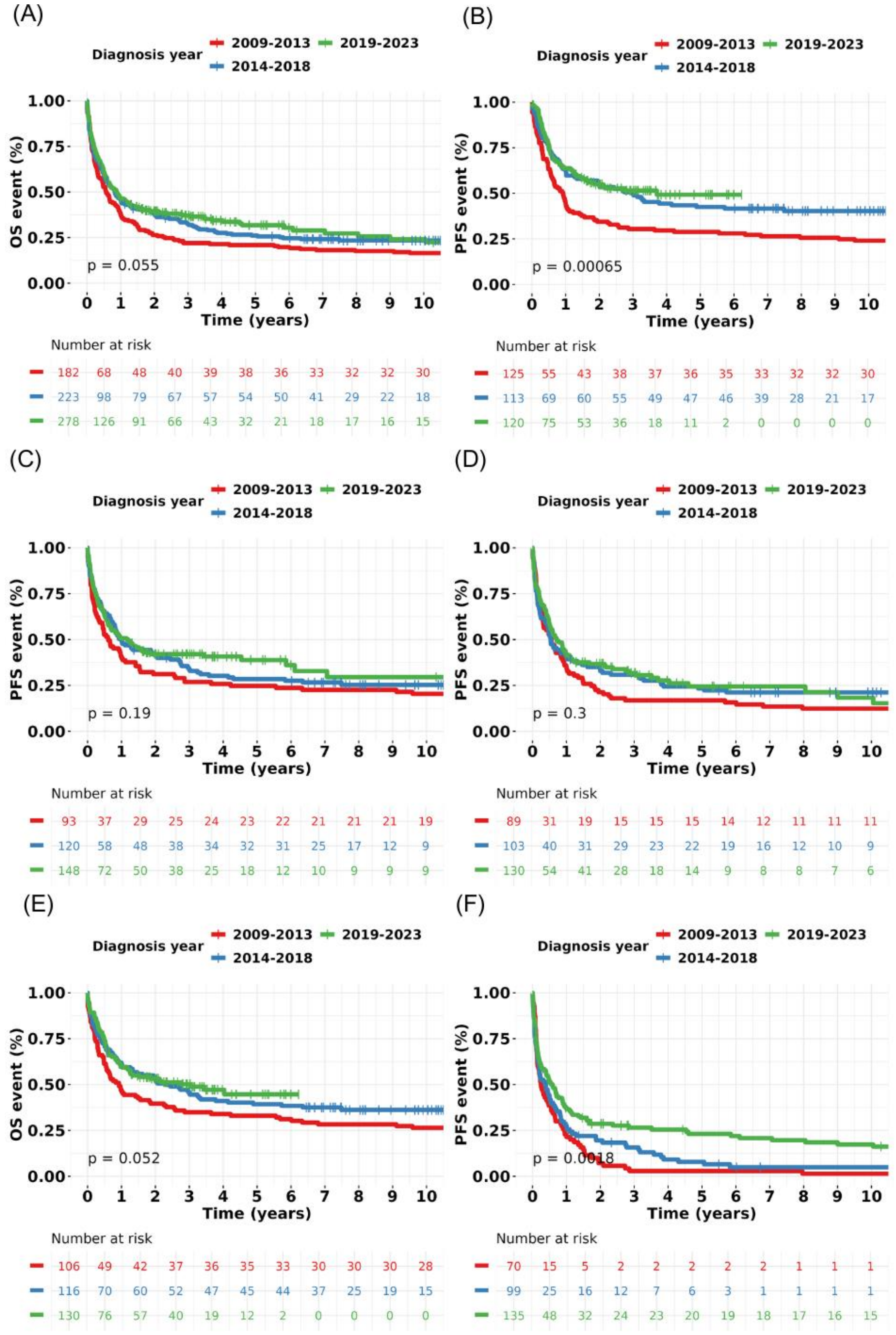

Supplementary Figure 5. Kaplan–Meier curve with log-rank p-value for overall survival in subgroups of acute myeloid leukemia.

(A) Female
(B) Male
(C) Age 16–69 years
(D) Age ≥70 years

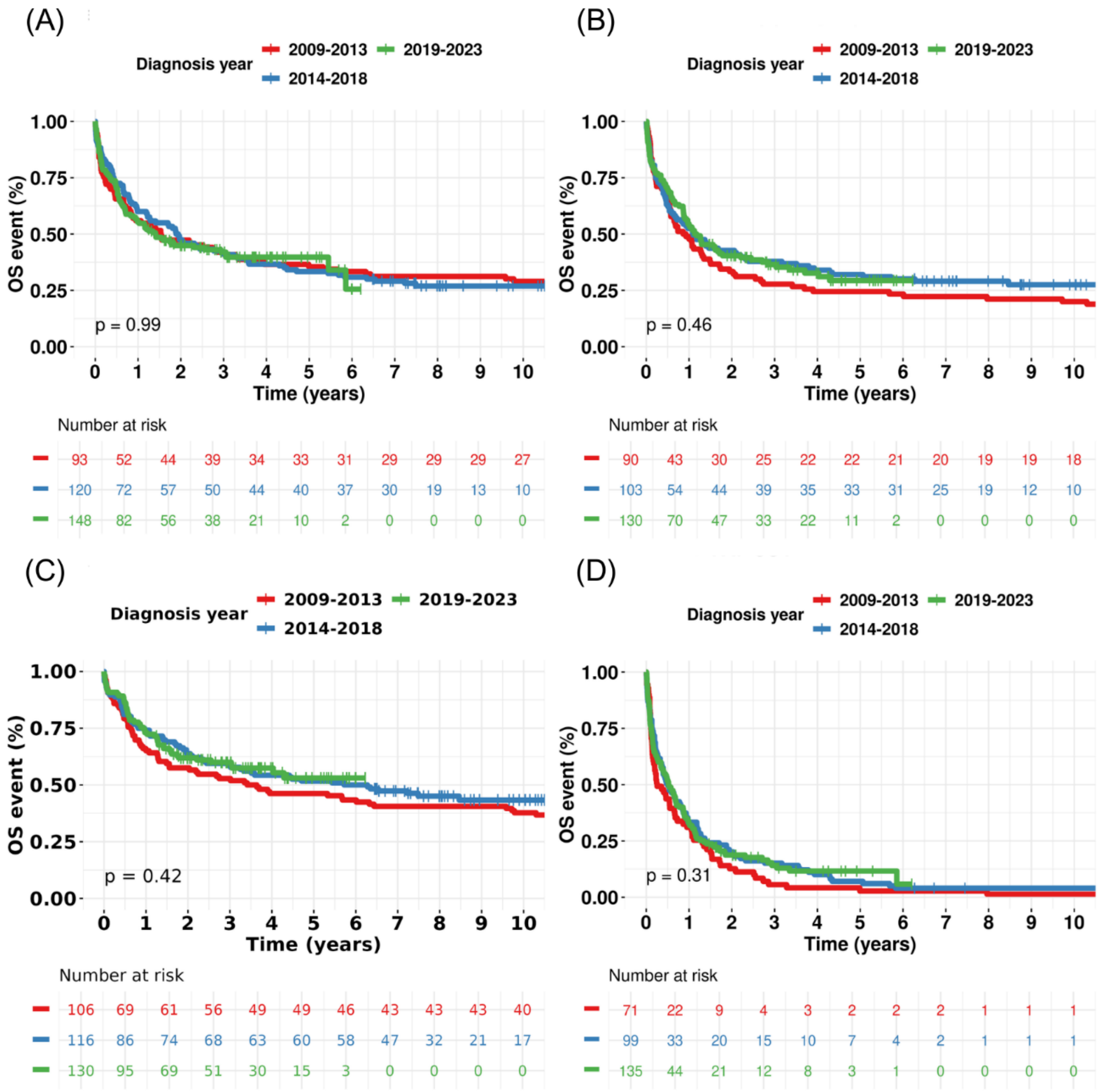

Supplementary Figure 6. Kaplan–Meier curve with log-rank p-value for overall survival in subgroups of myelodysplastic syndrome.

(A) Female
(B) Male
(C) Age 16–69 years
(D) Age ≥70 years

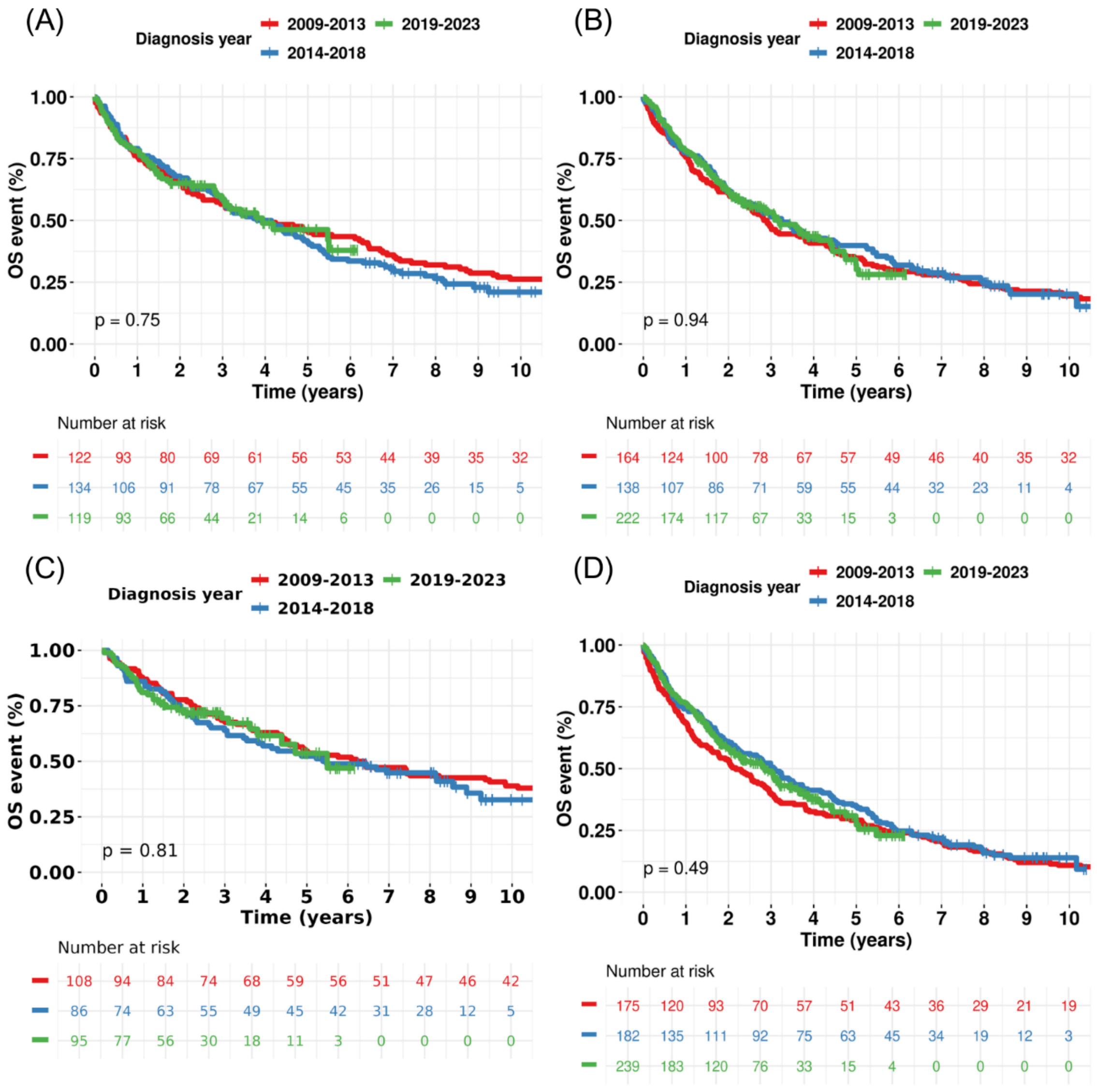

Supplementary Figure 7. Kaplan–Meier curve with log-rank p-value for overall survival in subgroups of myelofibrosis patients.

(A) Female
(B) Male
(C) Age 16–69 years
(D) Age ≥70 years

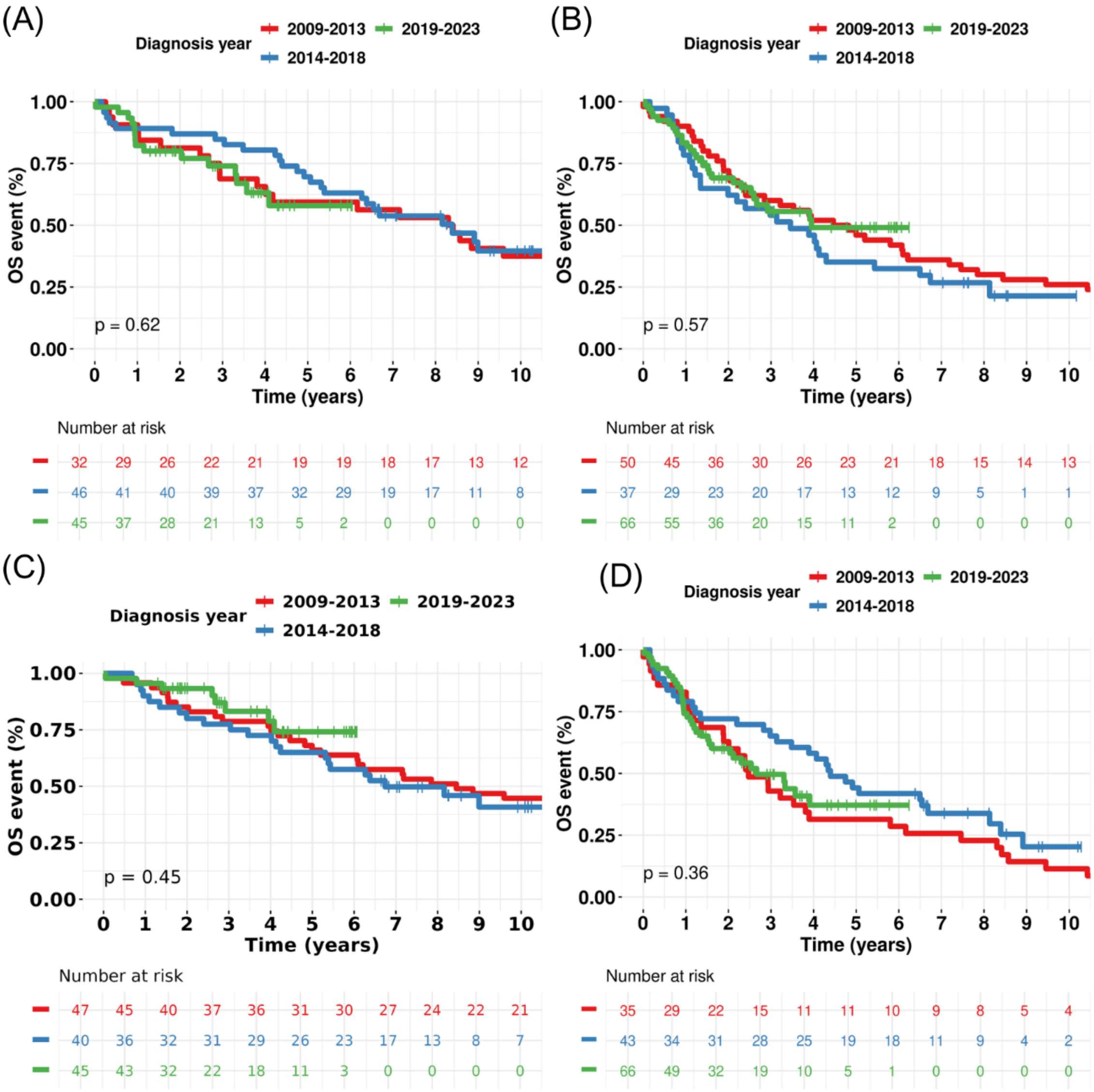

Supplementary Figure 8. Duration and number of hospital visits.

(A) Duration of inpatient visits (days) by diagnostic period
(B) Number of inpatient visits by diagnostic period

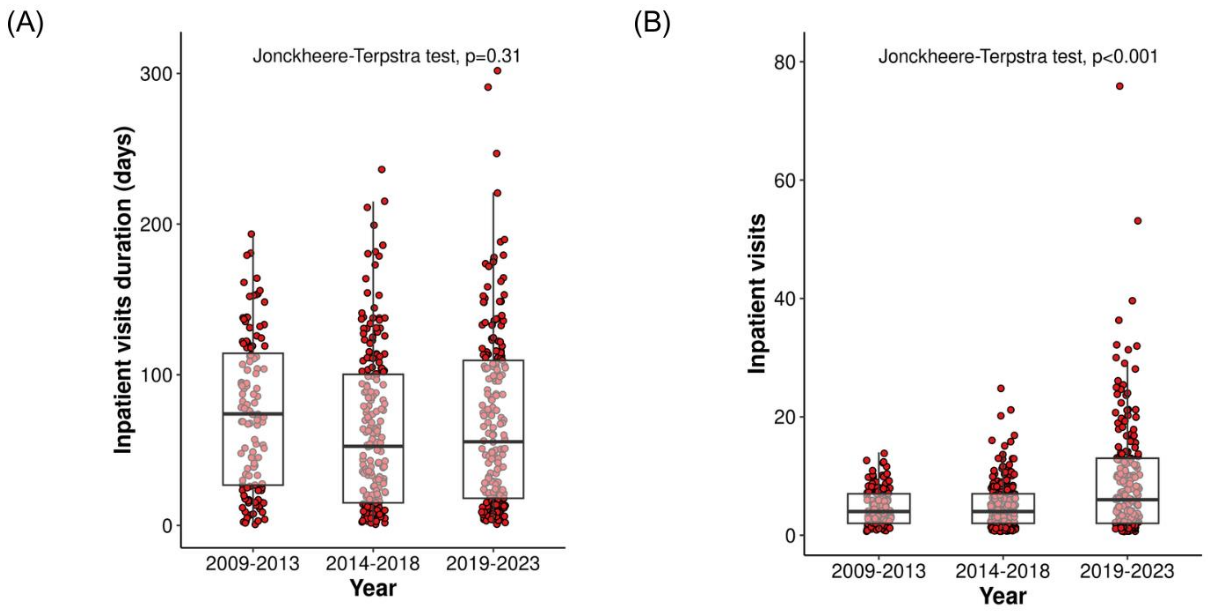

Supplementary Figure 9. Mean total medical cost in subgroups of risk stratification.

(A) Acute Myeloid Leukemia classified according to the 2022 European LeukemiaNet (ELN) risk stratification system.
(B) Myelodysplastic Syndromes classified using the Revised International Prognostic Scoring System (IPSS-R), dichotomized into higher-risk (IPSS-R > 3.5) and lower-risk (IPSS-R ≤ 3.5) groups.
(C) Myelofibrosis classified by the Dynamic International Prognostic Scoring System (DIPSS), grouped into higher-risk (high and intermediate-2) and lower-risk (intermediate-1 and low) categories.

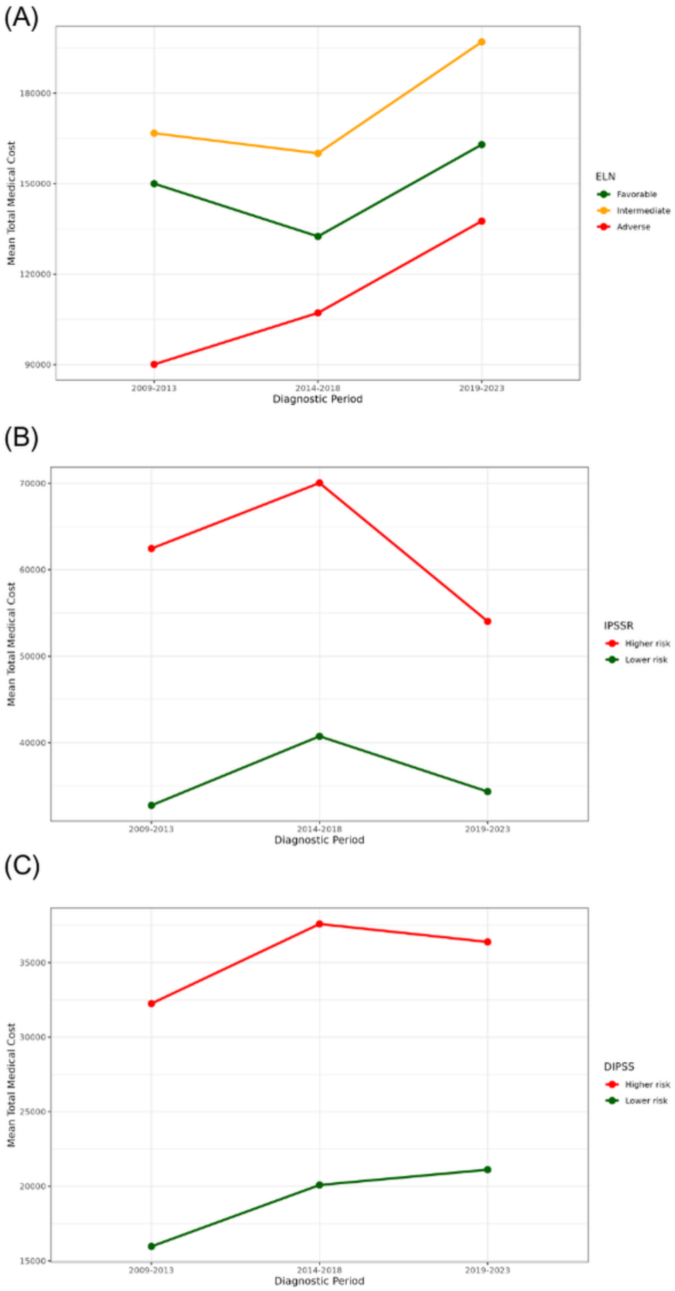

Supplement: Supplementary file 1 — Supplementary Material 1 [file 277_2026_6863_MOESM1_ESM.pdf]
